# Comparison of Serum Calcium, Phosphorus, Alkaline Phosphatase, and Intact Parathyroid Hormone Levels Between Dialysis-Dependent and Non-Dialysis-Dependent Patients With Chronic Kidney Disease: A Cross-Sectional Observational Study

**DOI:** 10.7759/cureus.116641

**Published:** 2026-09-21

**Authors:** Mustaq Ahmad, Ajesh Chandra Gupta, Yuvraj Gulati, Jailendra Kumar Gond, Mubashshir Qamar

**Affiliations:** 1 Department of Medicine, Ganesh Shankar Vidyarthi Memorial Medical College, Kanpur, IND; 2 Department of Nephrology, Ganesh Shankar Vidyarthi Memorial Medical College, Kanpur, IND; 3 Department of General Medicine, Ganesh Shankar Vidyarthi Memorial Medical College, Kanpur, IND

**Keywords:** chronic kidney disease, dialysis-dependent kidney disease, intact parathyroid hormone, mineral and bone disorder, mineral metabolism

## Abstract

Background

Chronic kidney disease (CKD) is frequently accompanied by chronic kidney disease-mineral and bone disorder (CKD-MBD), a systemic complication characterised by biochemical abnormalities in serum calcium, phosphorus, alkaline phosphatase (ALP), and intact parathyroid hormone (iPTH), which may contribute to bone disease, vascular calcification, cardiovascular morbidity, and adverse clinical outcomes.

Aim

This study aimed to compare serum calcium, phosphorus, ALP, iPTH, and calcium-phosphorus product levels between dialysis-dependent and non-dialysis-dependent patients with CKD and to assess the associations of these biochemical parameters with dialysis duration.

Materials and methods

This hospital-based comparative observational study included 120 adults with CKD attending a tertiary care centre in Kanpur, India, comprising 60 dialysis-dependent and 60 non-dialysis-dependent patients aged 18-65 years with CKD stages 3-5. Patients were enrolled consecutively during the eight-month study period following Institutional Ethics Committee approval. Serum calcium, phosphorus, ALP, and iPTH values were obtained as part of routine clinical evaluation and retrieved from the patients’ hospital records. In dialysis-dependent patients, blood samples were collected before a scheduled hemodialysis session; in non-dialysis-dependent patients, values were obtained from routine clinical laboratory assessments. The calcium-phosphorus product (Ca × P) was calculated from the recorded calcium and phosphorus values. Statistical analysis was performed using IBM SPSS Statistics, version 26.0 (IBM Corp., Armonk, NY, USA), with p < 0.05 considered statistically significant.

Results

Serum calcium was significantly lower, whereas serum phosphorus, ALP, iPTH, and calcium-phosphorus product were significantly higher in dialysis-dependent than in non-dialysis-dependent patients with CKD (all p < 0.001). All dialysis-dependent patients had stage 5 CKD. Among dialysis-dependent patients, dialysis duration showed significant positive correlations with serum phosphorus, ALP, and iPTH and a significant negative correlation with serum calcium.

Conclusion

CKD-MBD abnormalities were more pronounced in dialysis-dependent than in non-dialysis-dependent patients with CKD. Longer dialysis duration was associated with lower serum calcium and higher serum phosphorus, ALP, and iPTH levels. These findings support regular surveillance of these biochemical parameters for timely recognition and management of CKD-MBD.

## Introduction

Chronic kidney disease (CKD) is a progressive disorder associated with abnormalities in fluid, electrolyte, acid-base, endocrine, and mineral metabolism [1]. CKD-mineral and bone disorder (CKD-MBD) is a major systemic complication characterised by disturbances in calcium, phosphorus, parathyroid hormone (PTH), vitamin D metabolism, bone turnover, and vascular or soft-tissue calcification [2]. Disturbed phosphorus metabolism may also contribute to vascular calcification through mechanisms involving calcium-containing calciprotein particles, particularly in patients receiving hemodialysis [3].

Declining glomerular filtration reduces renal phosphate excretion, leading to phosphorus retention and progressive disturbances in mineral homeostasis [4]. Biochemical and hormonal indicators of CKD-MBD include serum calcium, phosphorus, alkaline phosphatase (ALP), intact parathyroid hormone (iPTH), and emerging markers such as fibroblast growth factor 23 (FGF23) [5]. Comparative evidence indicates that mineral metabolism abnormalities are generally more pronounced in dialysis-dependent than in non-dialysis-dependent patients with advanced CKD [4]. Changes in markers such as FGF23 and PTH may appear early during CKD progression and reflect evolving abnormalities in mineral regulation [6-7]. Increased bone turnover associated with secondary hyperparathyroidism may also be reflected by elevated serum ALP [8].

Abnormalities in serum calcium, phosphorus, and PTH become increasingly frequent with declining renal function [9]. Indian hospital-based evidence has also demonstrated a substantial burden of CKD-MBD, supporting the clinical importance of evaluating mineral abnormalities in this population [10]. Despite available management strategies, control of mineral metabolism may be influenced by differences in treatment, patient-related factors, and clinical characteristics [11]. Prolonged renal failure and secondary hyperparathyroidism are also associated with disturbances in vascular and mineral homeostasis [12]. Reduced calcitriol activity and altered hormonal regulation contribute to hypocalcaemia and progressive PTH stimulation as renal function deteriorates [13]. High serum phosphorus and calcium-phosphorus product levels have been associated with adverse outcomes in patients undergoing hemodialysis [14]. Persistent stimulation of the parathyroid glands may ultimately result in sustained secondary hyperparathyroidism and abnormal bone turnover [15].

Existing evidence establishes that CKD-MBD abnormalities become more prominent with advancing renal dysfunction, particularly among dialysis-dependent patients. Direct comparative assessment of serum calcium, phosphorus, ALP, iPTH, and calcium-phosphorus product within the same Indian tertiary care population remains clinically relevant, particularly because dialysis exposure and patient characteristics may influence biochemical profiles. The present study therefore compares these biochemical parameters between dialysis-dependent and non-dialysis-dependent patients with CKD and evaluates their association with dialysis duration.

Objective of the study

This study aimed to compare serum calcium, phosphorus, ALP, iPTH, and calcium-phosphorus product levels between dialysis-dependent and non-dialysis-dependent patients with CKD. It also assessed the association between dialysis duration and these biochemical parameters.

## Materials and methods

Study design

The present study was a hospital-based cross-sectional comparative observational study evaluating biochemical markers of mineral metabolism in dialysis-dependent and non-dialysis-dependent patients with CKD. The study was conducted over a period of eight months following approval from the Institutional Ethics Committee. The reporting of the study was guided by the Strengthening the Reporting of Observational Studies in Epidemiology (STROBE) recommendations.

Study setting

The study was carried out in the Department of Medicine, KPS Post Graduate Institute of Medicine, Ganesh Shankar Vidyarthi Memorial (GSVM) Medical College, Kanpur, Uttar Pradesh, India. Patients were recruited from Lala Lajpat Rai (LLR) Hospital and associated hospitals, including the medicine OPD, inpatient wards, and dialysis unit. The institute functions as a tertiary care centre serving both urban and rural patients with CKD. The study was conducted over eight months following Institutional Ethics Committee approval. Dialysis-dependent participants were patients receiving regular maintenance hemodialysis for at least three months, and dialysis status, frequency, and duration were verified from dialysis-unit records.

Study duration

After receiving approval from the Institutional Ethics Committee, the study was carried out over eight months, from August 2025 to March 2026.

Study population

Adult patients aged 18-65 years with CKD stages 3-5 were considered eligible for inclusion. CKD was diagnosed according to the Kidney Disease: Improving Global Outcomes (KDIGO) clinical practice guideline and was defined as an abnormality of kidney structure or function, including kidney damage or an estimated glomerular filtration rate (eGFR) of <60 mL/min/1.73 m², persisting for at least three months [16]. Chronicity was verified from the patients’ clinical history and available medical records documenting kidney disease or reduced renal function for at least three months. CKD diagnosis and staging were confirmed using clinical records and documented laboratory data. Participants were divided into two groups according to dialysis status. The dialysis-dependent CKD group comprised patients receiving regular maintenance hemodialysis for at least three months. The non-dialysis-dependent CKD group comprised patients with CKD stages 3-5 who were not receiving any form of renal replacement therapy at enrolment. Dialysis status, frequency, and duration were verified using dialysis-unit records. The selection and interpretation of serum calcium, phosphorus, ALP, and iPTH as CKD-MBD markers were based on the KDIGO CKD-MBD guidelines [17,18].

Sample size

A total of 120 patients were included in the study, comprising 60 dialysis-dependent CKD patients and 60 non-dialysis-dependent CKD patients. The sample size was estimated for comparison of biochemical parameters between the two independent groups using assumptions derived from previously published comparative evidence on mineral metabolism in dialysis-dependent and non-dialysis-dependent CKD patients [4]. The sample size was calculated for comparison of two independent means using the formula: n (per group) = 2 × (Zα/2 + Zβ)² × σ²/d², where Zα/2 = 1.96 for a 95% confidence level, Zβ = 0.84 for 80% statistical power, σ represents the pooled SD of the biochemical parameter, and d represents the expected mean difference between the two groups. Based on these assumptions, the minimum estimated sample size was approximately 52 participants per group. The final sample size was rounded to 60 participants per group, resulting in a total of 120 patients.

Sampling method

Consecutive sampling was used for participant recruitment. All eligible CKD patients attending the outpatient department, admitted to the medicine wards, or receiving care in the dialysis unit during the study period were screened. Patients who fulfilled the inclusion criteria and did not meet any exclusion criteria were enrolled consecutively until the required sample size was achieved. Both groups were recruited during the same study period using the same predefined eligibility criteria and consecutive sampling approach. No randomisation or matching was performed, and all eligible enrolled patients were included in the final analysis.

Inclusion criteria

Adult patients aged 18-65 years of either sex were eligible for inclusion. Patients were required to have CKD stages 3, 4, or 5 diagnosed and staged according to the KDIGO eGFR-based criteria described above [16]. Dialysis-dependent CKD patients were required to have received regular maintenance hemodialysis for at least three months. Non-dialysis-dependent CKD patients were required to have stage 3-5 CKD and no history of renal replacement therapy. Only patients who provided written informed consent were included.

Exclusion criteria

Patients diagnosed with acute kidney injury were excluded from the study. Patients receiving medications known to affect bone metabolism, including bisphosphonates or corticosteroids, were also excluded. Pregnant and lactating women were not included. Patients with other metabolic bone diseases, including primary hyperparathyroidism or malignancy, were excluded. Patients admitted to the ICU during the study period were also excluded.

Data collection tools

Demographic information, clinical history, examination findings, CKD diagnosis and stage, dialysis status, dialysis duration, and relevant laboratory parameters were recorded using a predesigned structured study proforma. The required information was obtained from hospital diagnostic records, inpatient and outpatient case records, dialysis-unit records, and available laboratory reports. In dialysis-dependent patients, blood samples were collected before a scheduled hemodialysis session as part of routine clinical evaluation. Serum calcium, serum phosphorus, ALP, and iPTH values were obtained from laboratory investigations documented in the patients’ hospital records. Serum calcium was measured using standard automated biochemical analysers, serum phosphorus by routine laboratory methods, ALP by standardised enzymatic methods on automated analysers, and iPTH by immunoassay techniques in the institutional central laboratory. Serum calcium and phosphorus were recorded in milligrams per decilitre (mg/dL), ALP in international units per litre (IU/L), and iPTH in picograms per millilitre (pg/mL). The calcium-phosphorus product was calculated by multiplying the recorded serum calcium concentration by the recorded serum phosphorus concentration and was expressed as mg²/dL². All demographic, clinical, dialysis-related, and biochemical data were transferred to the study proforma and subsequently used for statistical analysis.

Statistical analysis

All data were checked for completeness, consistency, and accuracy before analysis. Statistical analysis was performed using IBM SPSS Statistics, version 26.0 (IBM Corp., Armonk, NY, USA). Continuous variables were assessed for their distribution. Normally distributed variables were summarised as mean ± SD and compared between the dialysis-dependent and non-dialysis-dependent CKD groups using the independent-samples t-test. Non-normally distributed variables were summarised as median and IQR and compared using the Mann-Whitney U test. Categorical variables were expressed as frequencies and percentages and compared using the Pearson chi-square test; Yates continuity correction was applied to 2 × 2 comparisons where appropriate. ORs with 95% CIs were reported where applicable. Spearman’s rank correlation coefficient (ρ) was used to assess associations between dialysis duration and biochemical parameters and between iPTH and serum calcium, phosphorus, and ALP. All statistical tests were two-sided, and a p-value < 0.05 was considered statistically significant.

## Results

Baseline characteristics

A total of 120 eligible patients with CKD were enrolled, comprising 60 dialysis-dependent and 60 non-dialysis-dependent patients, and all enrolled participants were included in the final analysis, with no loss after enrolment. Baseline characteristics assessed in the study included age, sex, CKD stage, socioeconomic status, and dialysis duration in the dialysis-dependent group. Overall, 50.8% of participants were men and 49.2% were women. The 51-60-year age group represented the largest proportion of participants (31.7%), followed by 31-40 years (25.0%) and 41-50 years (23.3%). Gender distribution was comparable between the two groups (p = 0.715) (Table 1).

**Table 1:** Baseline characteristics of the study participants. CKD: Chronic kidney disease.

Variable	Category	Number, n	Percentage (%)
Age group, years	18-30	3	2.5
	31-40	30	25
	41-50	28	23.3
	51-60	38	31.7
	>60	21	17.5
CKD group	Dialysis-dependent CKD	60	50
	Non-dialysis-dependent CKD	60	50
Total		120	100

Table 2 shows the comparison of gender distribution between the CKD groups. Male and female participants were similarly distributed across the dialysis-dependent and non-dialysis-dependent groups, with no statistically significant association between gender and dialysis status (χ²(1) = 0.133, OR = 0.82, 95% CI: 0.40-1.68, p = 0.715).

**Table 2:** Comparison of gender distribution between CKD groups. Gender distribution was compared using the Pearson chi-square test with Yates continuity correction. The OR represents the odds of being male in the dialysis-dependent group relative to the non-dialysis-dependent group. The value in parentheses following χ² represents the degrees of freedom. CKD: Chronic kidney disease; χ²: Chi-square statistic.

Gender	Dialysis-dependent CKD, n (%)	Non-dialysis-dependent CKD, n (%)	Total, n (%)	Test statistic	OR (95% CI)	p-value
Male	29 (48.3)	32 (53.3)	61 (50.8)	χ²(1) = 0.133	0.82 (0.40-1.68)	0.715
Female	31 (51.7)	28 (46.7)	59 (49.2)			
Total	60 (100.0)	60 (100.0)	120 (100.0)			

Association between CKD group and CKD stage

All dialysis-dependent patients had stage 5 CKD, whereas non-dialysis-dependent patients included those with stage 3 (41.7%), stage 4 (18.3%), and stage 5 (40.0%) CKD. Overall, stage 5 CKD accounted for 70.0% of the study population (Table 3).

**Table 3:** Association between CKD group and CKD stage. CKD: Chronic kidney disease.

CKD stage	Dialysis-dependent CKD (n = 60)	Non-dialysis-dependent CKD (n = 60)	Total
Stage 3	0	25	25
Stage 4	0	11	11
Stage 5	60	24	84
Total	60	60	120

Association between CKD group and socioeconomic status

The distribution of socioeconomic status did not show a statistically significant association with CKD group (p = 0.509). Among the total participants, the lower socioeconomic class accounted for 34.2%, followed by the upper class at 33.3% and the middle class at 32.5%. The distribution was comparable between dialysis-dependent and non-dialysis-dependent CKD patients (Table 4).

**Table 4:** Association between CKD group and socioeconomic status. The association between socioeconomic status and CKD group was assessed using the Pearson chi-square test. The value in parentheses following χ² represents the degrees of freedom. CKD: Chronic kidney disease; χ²: Chi-square statistic.

Socioeconomic status	Dialysis-dependent CKD (n = 60)	Non-dialysis-dependent CKD (n = 60)	Total	Test statistic	p-value
Lower	22	19	41	χ²(2) = 1.350	0.509
Middle	21	18	39		
Upper	17	23	40		
Total	60	60	120		

Duration of dialysis among dialysis-dependent CKD patients

Among dialysis-dependent CKD patients, the largest proportion had been receiving dialysis for 13-24 months (31.7%), followed by more than 24 months (28.3%). Patients with a dialysis duration of 6-12 months accounted for 25.0%, while patients with less than six months of dialysis accounted for 15.0%. Overall, most dialysis-dependent patients had been on dialysis for more than one year (Table 5).

**Table 5:** Distribution of dialysis-dependent CKD patients according to dialysis duration. CKD: Chronic kidney disease.

Duration of dialysis	Number	Percentage (%)
<6 months	9	15
6-12 months	15	25
13-24 months	19	31.7
>24 months	17	28.3
Total	60	100

Correlation of dialysis duration with biochemical markers

The duration of dialysis showed statistically significant correlations with biochemical parameters. Serum calcium demonstrated a moderate negative correlation with dialysis duration (ρ = -0.312, p = 0.015), indicating lower calcium levels with increasing dialysis duration. Serum phosphorus (ρ = +0.428, p = 0.001), ALP (ρ = +0.462, p < 0.001), and iPTH (ρ = +0.518, p < 0.001) showed significant positive correlations with dialysis duration, indicating greater mineral metabolism abnormalities with longer dialysis duration (Table 6).

**Table 6:** Correlation of dialysis duration with biochemical markers in dialysis-dependent CKD patients. *Statistically significant. Spearman’s rank correlation test was used. Spearman’s ρ represents the correlation coefficient. CKD: Chronic kidney disease; iPTH: Intact parathyroid hormone; ALP: Alkaline phosphatase.

Variable	Spearman’s ρ	p-value
Dialysis duration versus serum calcium	-0.312	0.015*
Dialysis duration versus serum phosphorus	0.428	0.001*
Dialysis duration versus ALP	0.462	<0.001*
Dialysis duration versus iPTH	0.518	<0.001*

Comparison of vital parameters between CKD groups

The comparison of vital parameters between dialysis-dependent and non-dialysis-dependent CKD groups showed no statistically significant differences. Systolic blood pressure was slightly lower in the dialysis-dependent group, while diastolic blood pressure, pulse rate, and respiratory rate were comparable between the two groups. There was no discernible difference in haemodynamic status between the two CKD groups, as all measures had p-values greater than 0.05 (Table 7).

**Table 7:** Comparison of vital parameters between CKD groups. CKD: Chronic kidney disease; BP: Blood pressure.

Parameter	Dialysis-dependent CKD, median (IQR)	Non-dialysis-dependent CKD, median (IQR)	Standardized Z statistic	p-value
Systolic BP (mmHg)	147.0 (140.0-158.0)	153.0 (143.5-160.5)	-1.177	0.239
Diastolic BP (mmHg)	91.0 (86.0-94.0)	88.0 (84.0-94.0)	1.366	0.172
Pulse rate (beats/min)	84.0 (76.0-93.0)	87.0 (83.0-94.2)	-1.695	0.09
Respiratory rate (breaths/min)	19.0 (17.0-21.0)	18.5 (17.0-20.2)	1.071	0.284

Comparison of complete blood count parameters between CKD groups

The comparison of haematological parameters showed no statistically significant differences between dialysis-dependent and non-dialysis-dependent CKD groups. The dialysis-dependent group had slightly lower haemoglobin levels (10.04 ± 1.45 g/dL) than the non-dialysis-dependent group (10.37 ± 1.37 g/dL), but this difference was not statistically significant (p = 0.208). Total leukocyte count was also comparable between dialysis-dependent patients (7877 ± 1800) and non-dialysis-dependent patients (7785 ± 1629), with no significant difference observed (p = 0.770) (Table 8).

**Table 8:** Comparison of complete blood count parameters between CKD groups. CKD: Chronic kidney disease.

Parameter	Dialysis-dependent CKD, mean ± SD	Non-dialysis-dependent CKD, mean ± SD	Test statistic	p-value
Haemoglobin (g/dL)	10.04 ± 1.45	10.37 ± 1.37	t(118) = -1.281	0.208
Total leukocyte count (cells/mm³)	7877 ± 1800	7785 ± 1629	t(118) = +0.293	0.77

Comparison of kidney function test parameters between CKD groups

The dialysis-dependent CKD group showed significantly higher blood urea and serum creatinine levels compared with the non-dialysis-dependent CKD group. Blood urea was 120.5 mg/dL in the dialysis-dependent group compared with 81.5 mg/dL in the non-dialysis-dependent group, while serum creatinine was 8.3 mg/dL and 3.8 mg/dL, respectively. Both differences were statistically significant (p < 0.001), indicating poorer renal function among dialysis-dependent patients (Table 9).

**Table 9:** Comparison of kidney function test parameters between CKD groups. *Statistically significant. Variables were compared using the Mann-Whitney U test. CKD: Chronic kidney disease.

Parameter	Dialysis-dependent CKD, median (IQR)	Non-dialysis-dependent CKD, median (IQR)	p-value
Blood urea (mg/dL)	120.5 (92.5-141.2)	81.5 (58.8-108.0)	<0.001*
Serum creatinine (mg/dL)	8.3 (7.4-9.4)	3.8 (2.7-4.7)	<0.001*

Comparison of electrolyte parameters between CKD groups

The comparison of serum electrolyte levels showed no statistically significant differences between the dialysis-dependent and non-dialysis-dependent CKD groups. Serum sodium levels were comparable between the groups (139.0 ± 3.9 mEq/L vs 138.2 ± 3.9 mEq/L; p = 0.259). Serum potassium levels were slightly lower in the dialysis-dependent group than in the non-dialysis-dependent group, but this difference was not statistically significant (4.70 vs 5.00 mEq/L; p = 0.503), indicating similar electrolyte status between the two groups (Table 10).

**Table 10:** Comparison of electrolyte parameters between CKD groups. Serum sodium was compared using the independent-samples t-test. Serum potassium was compared using the Mann-Whitney U test. The standardized Z statistic for potassium was reconstructed from the reported two-sided p-value because the original U statistic was not retained in the archived summary data. The value in parentheses following t represents the degrees of freedom. CKD: Chronic kidney disease; t: t-statistic; Z: Standardized Z statistic.

Parameter	Dialysis-dependent CKD (n = 60)	Non-dialysis-dependent CKD (n = 60)	Test statistic	p-value
Serum sodium (mEq/L), mean ± SD	139.0 ± 3.9	138.2 ± 3.9	t(118) = +1.124	0.259
Serum potassium (mEq/L), median (IQR)	4.70 (4.20-5.17)	5.00 (4.10-5.40)	Z = −0.670	0.503

Comparison of biochemical parameters and CKD-MBD categories

The dialysis-dependent CKD group showed significantly more pronounced abnormalities in mineral metabolism than the non-dialysis-dependent CKD group. Serum calcium was significantly lower among dialysis-dependent patients, whereas serum phosphorus, ALP, iPTH, and calcium-phosphorus product were significantly higher in this group. A greater proportion of dialysis-dependent patients had low serum calcium (43.3%), high serum phosphorus (100.0%), high ALP (100.0%), high iPTH (100.0%), and elevated calcium-phosphorus product (51.7%) compared with non-dialysis-dependent patients. These findings represent unadjusted between-group differences and should not be interpreted as an independent effect of dialysis because all dialysis-dependent patients had stage 5 CKD, whereas the non-dialysis-dependent group included patients with stages 3-5 CKD (Table 11).

**Table 11:** Comparison of biochemical parameters and their categories between dialysis-dependent and non-dialysis-dependent CKD patients. *Statistically significant. CKD: Chronic kidney disease; n: Number of patients; mg/dL: Milligrams per decilitre; IU/L: International units per litre; iPTH: Intact parathyroid hormone; pg/mL: Picograms per millilitre; mg²/dL²: Milligrams squared per decilitre squared; ALP: Alkaline phosphatase.

Parameter	Dialysis-dependent CKD (n = 60)	Non-dialysis-dependent CKD (n = 60)	Test statistic	Effect estimate	p-value
Serum calcium (mg/dL), median (IQR)	8.52 (8.18-8.95)	9.07 (8.76-9.52)	Z < −3.291	Not applicable	<0.001*
Serum phosphorus (mg/dL), median (IQR)	6.25 (5.68-6.92)	4.20 (3.81-4.55)	Z > +3.291	Not applicable	<0.001*
ALP (IU/L), mean ± SD	306.36 ± 81.48	155.59 ± 32.40	t(118) = 13.319	Cohen’s d = 2.43	<0.001*
iPTH (pg/mL), median (IQR)	736.50 (586.75-942.25)	232.50 (144.25-316.75)	Z > +3.291	Not applicable	<0.001*
Calcium-phosphorus product (mg²/dL²), mean ± SD	53.8 ± 6.8	38.1 ± 4.2	t(118) = 15.216	Cohen’s d = 2.78	<0.001*
Low serum calcium, n (%)	26 (43.3)	11 (18.3)	χ²(1) = 7.659	OR = 3.41 (95% CI: 1.49-7.81)	0.006*
High serum phosphorus, n (%)	60 (100.0)	16 (26.7)	χ²(1) = 66.352	Corrected OR = 326.33 (95% CI: 19.07-5585.69)	<0.001*
High ALP, n (%)	60 (100.0)	52 (86.7)	χ²(1) = 6.563	Corrected OR = 19.59 (95% CI: 1.10-347.62)	0.010*
High iPTH, n (%)	60 (100.0)	18 (30.0)	χ²(1) = 61.575	Corrected OR = 277.97 (95% CI: 16.30-4740.27)	<0.001*
Elevated calcium-phosphorus product (≥55 mg²/dL²), n (%)	31 (51.7)	0 (0.0)	χ²(1) = 39.145	Corrected OR = 129.20 (95% CI: 7.64-2185.54)	<0.001*

Correlation of biochemical markers with serum iPTH levels

Correlation analysis showed no significant relationship between serum calcium, serum phosphorus, or ALP and iPTH levels within the dialysis-dependent and non-dialysis-dependent CKD groups individually. In the overall CKD population, serum calcium showed a significant negative correlation with iPTH, whereas serum phosphorus and ALP showed strong positive correlations with iPTH. These findings indicate greater CKD-MBD abnormalities with increasing biochemical imbalance among patients with CKD (Table 12).

**Table 12:** Correlation of biochemical markers with serum iPTH levels in CKD patients. *Statistically significant. Spearman’s rank correlation test was used. Spearman’s ρ represents the correlation coefficient. CKD: Chronic kidney disease; iPTH: Intact parathyroid hormone; ALP: Alkaline phosphatase.

Variable	Dialysis-dependent CKD, Spearman’s ρ	p-value	Non-dialysis-dependent CKD, Spearman’s ρ	p-value	Overall CKD, Spearman’s ρ	p-value
Serum calcium versus iPTH	-0.154	0.249	-0.005	0.968	-0.496	<0.001*
Serum phosphorus versus iPTH	-0.171	0.2	0.06	0.657	0.736	<0.001*
ALP versus iPTH	-0.213	0.108	-0.251	0.057	0.617	<0.001*

## Discussion

Dialysis-dependent CKD patients had significantly lower serum calcium levels and significantly higher serum phosphorus, ALP, iPTH, and calcium-phosphorus product than non-dialysis-dependent CKD patients (p < 0.001). These findings indicate a substantially greater biochemical burden of CKD-MBD in the dialysis-dependent group. Nevertheless, this difference should be interpreted in the context of CKD severity, as all dialysis-dependent participants had stage 5 CKD, whereas the non-dialysis-dependent group included stages 3-5 CKD. Thus, the observed biochemical differences likely reflect the combined effects of advanced renal dysfunction and dialysis dependence rather than an independent effect of dialysis alone. The lower calcium levels in dialysis-dependent patients are consistent with previous studies [11,12] showing that progressive CKD impairs renal calcitriol production, reduces intestinal calcium absorption, and promotes secondary hyperparathyroidism. Persistent phosphate retention may further reduce circulating calcium and contribute to continued stimulation of the parathyroid glands.

Serum phosphorus was markedly higher in dialysis-dependent patients. This finding is biologically plausible because declining glomerular filtration progressively limits renal phosphate excretion, and compensatory phosphaturic mechanisms become insufficient in advanced CKD. Similar findings have been reported in previous studies [13,14], which identified phosphorus retention as a characteristic abnormality of end-stage renal disease and an important contributor to vascular calcification and cardiovascular risk. The coexistence of higher phosphorus and lower calcium provides a physiological stimulus for increased PTH secretion and may promote progressive disturbances in bone turnover. The higher calcium-phosphorus product observed in the dialysis-dependent group further indicates a biochemical environment associated with increased calcification risk, although clinical vascular calcification was not directly assessed in the present study.

A pronounced difference in iPTH levels was also observed between the groups, supporting the presence of more marked secondary hyperparathyroidism among dialysis-dependent patients. Previous reports [15,19] similarly demonstrated progressive increases in PTH as renal function declines, particularly in the presence of hypocalcaemia, phosphate retention, and impaired vitamin D activation. Persistent PTH stimulation can increase skeletal turnover, which may partly explain the substantially higher ALP levels observed in the dialysis-dependent group. These findings are consistent with previous data [20] linking elevated ALP and disturbed calcium-phosphorus metabolism with increased bone turnover and vascular calcification in advanced CKD. From a clinical perspective, simultaneous assessment of calcium, phosphorus, iPTH, and ALP is important because no single biochemical marker adequately represents the complex and interrelated abnormalities of CKD-MBD. Maintaining appropriate mineral balance remains central to reducing the potential skeletal and cardiovascular consequences of CKD-MBD [21].

The correlation findings provide further insight into these relationships. In the overall CKD population, serum calcium was negatively correlated with iPTH, whereas serum phosphorus and ALP were positively correlated with iPTH. This pattern is physiologically consistent with secondary hyperparathyroidism: declining calcium and increasing phosphate contribute to PTH stimulation, and sustained PTH excess is accompanied by increased bone turnover reflected by ALP. These findings are comparable with previous studies [22] reporting increasing PTH concentrations alongside worsening biochemical abnormalities in CKD. In contrast, the absence of statistically significant correlations within the dialysis-dependent and non-dialysis-dependent groups suggests that the strong overall associations may partly reflect differences between disease-severity groups rather than uniform relationships within each group. Restricted variability after stratification and the relatively small number of participants in each subgroup may also have reduced the ability to detect significant within-group correlations.

The findings therefore support the established pathophysiological progression of CKD-MBD, in which declining renal function is accompanied by phosphate retention, impaired calcium and vitamin D homeostasis, secondary hyperparathyroidism, and altered bone turnover. The substantially greater biochemical abnormalities in dialysis-dependent patients identify this group as having a higher CKD-MBD burden, but the stage imbalance between groups limits attribution of these abnormalities specifically to dialysis. Accordingly, the findings support regular biochemical surveillance and integrated interpretation of calcium, phosphorus, ALP, and iPTH, particularly in patients with advanced CKD.

Limitations and future recommendations

This study has several limitations. Its single-centre, hospital-based observational design and relatively modest sample size may limit the generalisability and statistical precision of the findings. The dialysis-dependent group consisted entirely of stage 5 CKD patients, whereas the non-dialysis-dependent group included stages 3-5 CKD, resulting in substantial confounding by disease severity; therefore, the observed biochemical differences cannot be attributed independently to dialysis status. A stage 5-specific comparison was not performed, further limiting separation of the effects of dialysis from those of advanced CKD. The cross-sectional design also prevents causal or longitudinal interpretation. In addition, total ALP is not specific to bone turnover, and the absence of serum albumin and albumin-corrected calcium may affect interpretation of calcium status. Important factors, including vitamin D status, dietary phosphate intake, FGF23, residual renal function, dialysis adequacy, dialysate calcium concentration, and medication use, were not evaluated.

Future multicentre prospective studies should include larger, stage-matched cohorts, detailed treatment and dialysis data, serial biochemical measurements, and adjustment for relevant confounders. Incorporating imaging, vascular calcification assessment, and clinical outcomes would provide a more comprehensive evaluation of CKD-MBD.

## Conclusions

The present study found that dialysis-dependent CKD patients had lower serum calcium and higher serum phosphorus, ALP, iPTH, and calcium-phosphorus product than non-dialysis-dependent patients. These findings indicate a greater biochemical burden of CKD-MBD in the dialysis-dependent group, although this difference should be interpreted in the context of more advanced CKD stage. Longer dialysis duration was associated with lower serum calcium and higher serum phosphorus, ALP, and iPTH levels. These findings support periodic monitoring of serum calcium, phosphorus, ALP, and iPTH in patients with advanced CKD to facilitate timely recognition and management of CKD-MBD.
